# Driving behavior characterization and traffic emission analysis considering the vehicle trajectory

**DOI:** 10.3389/fpsyg.2023.1341611

**Published:** 2024-01-29

**Authors:** Xuejiao Du, Xiuyun Kang, Yan Gao, Xi Wang

**Affiliations:** ^1^College of Marxism, Northeast Normal University, Changchun, China; ^2^College of Marxism, Changchun University of Traditional Chinese Medicine, Changchun, China; ^3^Jilin Land Planning Research Office, Changchun, China; ^4^Institute of Economic Research, Jilin Academy of Social Sciences, Changchun, China

**Keywords:** driving behavior, K-means algorithm, symbol approximation aggregation, MOVES, traffic emissions

## Abstract

Based on the development of the concept of a resource-saving and environmentally friendly society, needing to develop low-carbon and sustainable urban transportation. Most of the pollutants come from the emissions of motor vehicle exhaust. Therefore, this paper analyzes the relationship between driving behavior and traffic emissions, to constrain driver behavior to reduce pollutant emissions. The GPS data are preprocessed by using Navicat for data integration, data screening, data sorting, etc., and then, the speed data are cleaned by using a combination of box-and-line plots and linear interpolation in SPSS. Second, this paper uses principal component analysis (PCA) to downsize 12 indicators such as average speed, average acceleration, and maximum speed and then adopts K-MEANS and K-MEDOIDS methods to cluster the driver’s behavioral indicators, selects the aggregation method based on the clustering indexes optimally, and analyzes the driver’s driving state by using the symbolic approximation aggregation method; finally, according to the above research results and combined with the MOVES traffic emission model to analyze the relationship between the driver’s driving mode, driving state, and traffic emissions, the decision tree can be used to predict the unknown driving mode of the driver to estimate the degree of its emissions.

## 1 Introduction

**Figure 1:**
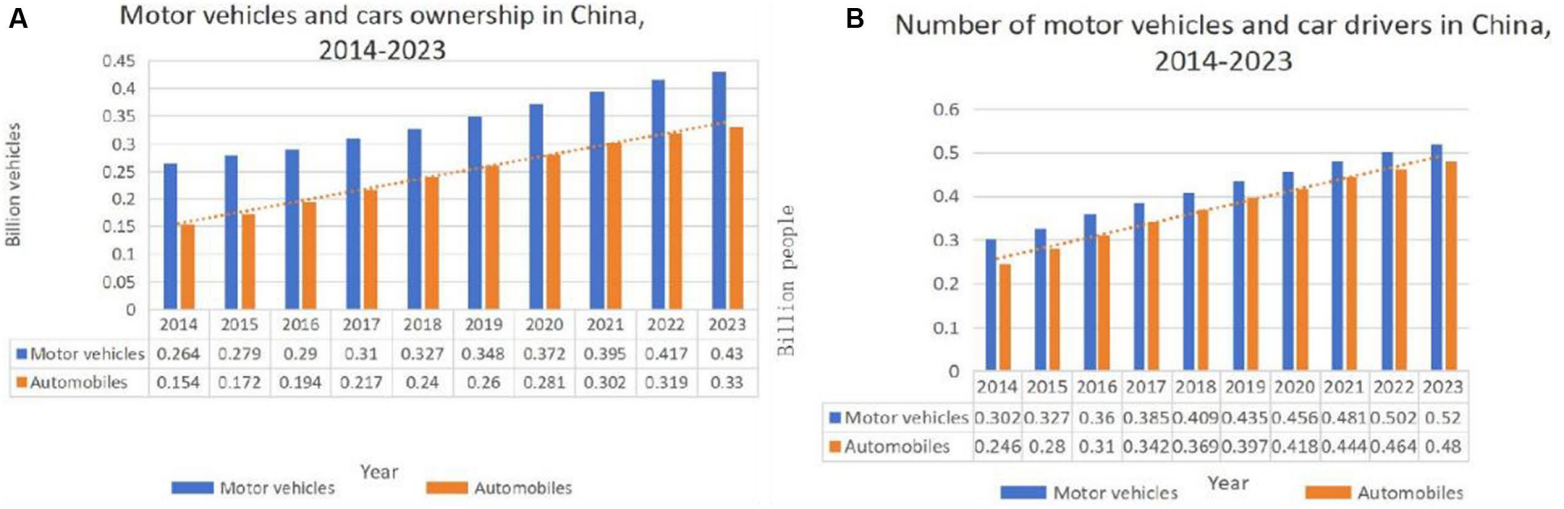
Development status of motor vehicles from 2014 to 2023, **(A)** motor vehicles and vehicle ownership; **(B)** motor vehicles and their drivers.

With the rapid economic and social development in China, the number of motor vehicles is growing at a high speed, and the resulting traffic emission problem has become a major problem affecting the ecological environment. Tailpipe emissions, as a by-product of modern transportation travel, not only affect human life and health but also lead to irreversible ecological problems such as tree dieback and crop yield reduction. Therefore, the study of vehicular traffic emissions has important theoretical significance and practical significance. At the same time, the number of motor vehicles is growing at a high speed, which has become a phenomenon that cannot be ignored. According to the statistics of the relevant departments, as of the first half of 2023, China’s motor vehicle ownership has exceeded 400 million, the following Figure 1A for the development of motor vehicles in recent years intuitively shows that the number of motor vehicles is growing quite fast, and cars accounted for a large proportion of motor vehicles, almost linear growth.

In addition, while the number of motor vehicles in China continues to increase, the number of motorists is also growing. The data in Figure 1B show that the number of drivers and the number of motor vehicles have been growing in a general trend, and the number of drivers is much higher than the number of motor vehicles.

Emissions from automobiles have always had a serious impact on the environment, which is contrary to the concept of sustainable development. Emissions produced by automobiles are both gaseous and particulate, and they are not only harmful to people’s health but also irreparably harm the environment. The three main vehicle emission species (carbon monoxide, hydrocarbons, and nitrogen oxides) have a significant impact on air pollution (Liu et al., 2022). According to the news released by the official microblog of the Ministry of Ecology and Environment, the Annual Report on Environmental Management of Mobile Sources in China (2022) showed that in 2021, the total emission of four pollutants from motor vehicles (including cars, three-wheeled vehicles, low-speed trucks, and motorcycles) in the country was 15.577 million tons. Based on the above statistics, it can be introduced that the air pollution problems will become more and more serious when the number of motor vehicles and the number of drivers grow in parallel (Lyu et al., 2021). There are various factors affecting the generation of motor vehicle exhaust, such as the type of motor vehicle, the type of fuel, and the driving behavior of drivers. The latest traffic emission model MOVES (Liu et al., 2019) will be divided into 13 types of motor vehicles, each type of motor vehicles, although in the same working conditions under the emission situation is not the same (Hao et al., 2017; Ramadan et al., 2022); for the type of automobile fuel, there are a variety of types of fuel, the public generally known as gasoline, diesel fuel, and natural gas, but also some cars use ethanol, methanol, and liquid hydrogen and in recent years gradually developed electric power, of which, natural gas is the most popular type, the gas is the most common. Some cars use ethanol, methanol, and liquid hydrogen and gradually developed electric power in recent years, among which natural gas, methanol, ethanol, liquid hydrogen, etc. are clean fuels with less air pollution (Guo et al., 2018).

GPS can acquire vehicle location information in real time (Shan et al., 2019), thus forming a vehicle trajectory in a continuous time series. Vehicle trajectory is both a performance feature in the operating behavior of the vehicle driver and is closely related to the tailpipe emissions of the vehicle at the same time. The emissions produced by different drivers’ driving patterns are different, and the proposed trajectory-based approach represents a microscopic method for estimating vehicle fuel consumption and tailpipe emissions, which can use individual vehicle trajectories as inputs to the emission model. At a fine level, the emissions of each vehicle can be estimated. In this paper, we mainly analyze driving behavior based on trajectory data, using machine learning as well as data analysis, and clustering the driver’s driving pattern based on real-time positioning data, as well as analyzing the driver’s driving state, both the macro-driving pattern and the micro-driving state have a direct impact on the tailpipe emissions of motor vehicles. It is of great significance to study the relationship between driving behavior and tailpipe emissions, so that driver behavior can be constrained to reduce the emission of pollutants.

GPS trajectory data are related to both driver behavior and also closely related to traffic emissions, with the wide popularity of cars, cell phones, and other smart products, GPS is developing rapidly and has a lot of space for development in many fields, and many scholars have done a lot of research on it. For example, Dabiri et al. (2020) proposed a novel GPS trajectory representation method using GPS data, which was not only compatible with deep learning models but also capable of capturing vehicle motion features and road features. Zhang et al. (2015) based on a large GPS history database of approximately 7,600 cabs in a Chinese city in 1 year, to understand the cab service strategy from cab GPS tracking. Lou and Cheng (2020) detected changes in individual travel behavior patterns based on GPS/GNSS. Pal and Chunchu (2018) proposed an adaptive noise smoothing method based on fully integrated empirical modal decomposition for trajectory data to remove noise from trajectory data and improve data utilization.

## 2 Literature review

The following is specific to scholars’ research on driving behavior and traffic emissions.

### 2.1 Current status of driving behavior research

Drivers produced a variety of behaviors during driving, such as speeding, acceleration and deceleration, idling, and parking. In driver-related research, most of the studies were centered on the relationship between drivers’ driving behaviors and traffic safety or risky driving. For example, An et al. used a risk prediction model of driving behavior based on non-linear support vector product to predict the risk level of driving behavior using multiple indicators, and it was able to effectively predict the state of drivers and vehicles (An et al., 2021); Constantinescu et al. investigated a model of different drivers’ driving styles based on several driving parameters (speed, acceleration, braking, etc.), which could help any driver to see the dangers in his driving style (Constantinescu et al., 2010); Sun et al. (2019) derived a variety of driving behavior determination methods based on SOM and other methods and obtained some driving behavior determination methods for complex road sections; Sun et al. (2015) proposed a combination of factor analysis and systematic clustering with the help of SPSS software to cluster the driver’s behavioral characteristics and thus derive the driver category with higher driver categories with higher driving risks.

Some researchers have studied elderly drivers who make up a relatively small percentage of drivers. Zhao et al. (2018) explored the driving behaviors of senior drivers, including road choice, left/right turns, and driving speed. The road choice analysis showed that senior drivers were reluctant to get on the freeway not only for a short period but also for a long period. This study did not find a significant difference between senior drivers and non-senior drivers when turning at intersections. To explore the influencing factors of driving speed, a random-effects regression model was constructed with age, gender, road type, and the interaction term between age and road type as explanatory variables (Zhao et al., 2018). Some researchers have also investigated the relationship between driving environments and driving behaviors, and Marta identified several driving behavior patterns of driving environments (based on street level and weather conditions) from real-environment driving data and analyzed how these driving environments affect driving behavior (Faria et al., 2020). Most measures of driving behavior have focused on categorization, while few studies have focused on individual driving behavior characteristics. However, it was also important for drivers to identify and correct their risky behaviors and optimize their driving. Therefore, Chen et al. proposed a graphical method to simulate individual driving behavior for driving safety analysis (Chen et al., 2019). Some studies use drivers’ driving behaviors to analyze their relationship with traffic emissions. For example, Zheng et al. (2017) subjectively classified drivers based on a questionnaire to investigate the relationship between their behavioral characteristics and traffic emissions. However, this approach was subjective and lacked a theoretical basis. Sun et al. estimated drivers’ emissions at intersections based on their driving behavior at intersections (Gu, 2020). Although it can estimate the emission at intersections more accurately, the emission process was during the whole driving process and ignores the emission process of road sections (Sun et al., 2015).

### 2.2 Current status of transportation emission research

The MOVES model used specific power to effectively combine motor vehicle instantaneous emission rates and operating conditions. Based on the idea of coupling CarSim, TruckSim, and MOVES and localizing the parameters that needed to be inputted, Gu mainly investigated the relationship between road gradient and pollutant emissions and established an emission prediction model for different gradients (Gu, 2020). Lin et al. (2019) added the GPS data into a modified MOVES model to improve emission accuracy; Zoe and Naomi (2021) used MOVES and explored changes in fleet emissions due to the proliferation of connected and automated vehicles (CAVs) in the Vancouver metropolitan area in the 2030 and 2040 timeframes. Perugu modified the MOVES model to incorporate a local light-duty vehicle-specific driving cycle in India to modify the emission rates (Perugu, 2019). Yao et al. (2019) localized the main input parameters of the MOVES model and obtained an emission inventory for the city. Ravindra estimated the total emissions during vehicle idling and validated the emission results from real data (Kumar et al., 2015).

In addition to this, some studies did not use the above-mentioned emission models. For example, Sofwan and Latif (2020) used an exhaust analyzer to obtain pollutant emissions and then used hierarchical cluster analysis to examine the effect of actual driving conditions on tailpipe emissions. Pavlovic et al. (2021) analyzed the accuracy, vehicle measurement distance, and specific fuel consumption rate of on-board fuel consumption measurement systems installed in light-duty trucks and heavy-duty trucks. Ping et al. (2019) obtained instantaneous fuel consumption using ECUs. Kanarachos et al. (2019) used smartphones and recurrent neural networks to estimate instantaneous vehicle fuel consumption. Sergio used GPS to predict fuel consumption and emissions based on traffic flow using a vehicle emissions inventory model (VEN) (Ibarra-Espinosa et al., 2020).

Most of the existing studies on vehicle traffic emissions are based on objective data for simulation and optimization of traffic emissions, traffic emission influencing factors, etc., with little consideration of human subjective initiative. In this paper, from the human subjective initiative, we consider the driving behavior of drivers, based on a number of indicators of driving behavior, combined with the MOVES aligned emission model, innovatively use machine learning methods to cluster analysis of the driver’s driving pattern, and then analyze the relationship between driving behavior and emissions.

Based on this, the research significance of this paper is mainly reflected in the following aspects:

1. Considering the subjective initiative of human beings, the relationship between driving behavior and tailpipe emissions is investigated with a view to reducing tailpipe emissions by restraining drivers’ driving behavior;
2. cluster analysis of drivers’ driving patterns, defining different drivers’ driving patterns, and providing a detailed theoretical basis for future research on related aspects;
3. propose corresponding countermeasures through the results of the study, with a view to reducing motor vehicle tailpipe emissions and environmental pollution, so as to develop low-carbon and energy-saving urban transportation, which is of great practical significance to the local superior government in reducing emission pollution.

## 3 Data analysis

### 3.1 Data source

This paper utilizes GPS data from the city of A from 2 September to 30 September 2023, totaling more than 15 million entries, and each data contain driver number, time, speed, latitude, longitude, etc. GPS data field descriptions and examples are shown in Supplementary Table S1: Research manuscripts reporting large datasets that are deposited in a publicly available database should specify where the data have been deposited and provide the relevant accession numbers. If the accession numbers have not yet been obtained at the time of submission, please state that they will be provided during review. They must be provided before publication.

### 3.2 Driving behavior analysis based on GPS data

#### 3.2.1 Analysis of speed fluctuation

**Figure 2:**
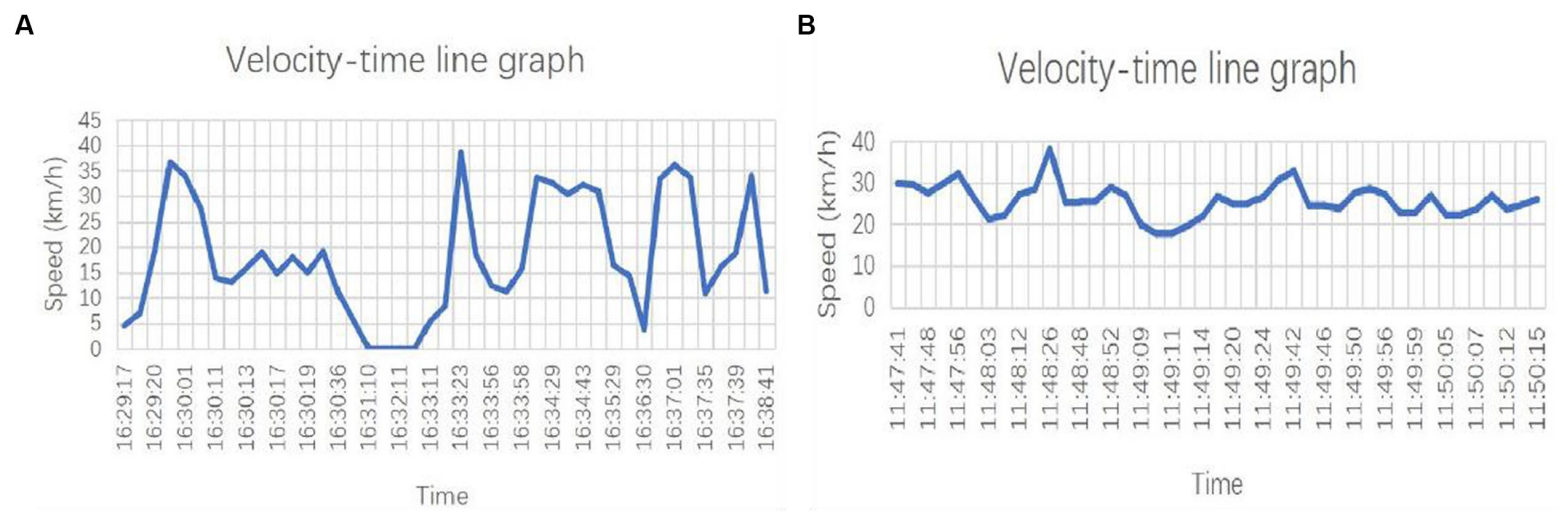
Draw the speed time line diagram according to the time series. **(A)** driver 1 speed time line diagram; **(B)** driver 2 speed time line chart.

From the data obtained from the GPS data, it can be found that in all data items, the speed is the most enough to directly reflect the driving behavior; according to the time-series data, the speed timeline graph can be drawn in Figure 2A, which can be visualized to see the speed of change. Take the time-series sequence of a driver as an example, here the driver is named driver 1, in 16:31:10 moments from the time, the speed of its continuous 0, it can be assumed that the driver at this time on the way to the intersection is stopping and waiting, and then start to leave. In addition, from the whole sequence, it can be intuitively observed that the driver’s speed fluctuation is large, the maximum fluctuation is 30 km/h within 10s, its acceleration is 0.84 m/s2, and acceleration and deceleration are obvious.

Figure 2B shows the velocity line graph of another driver, named Driver 2. This graph reflects the waveform of the driver’s velocity in the unstopped state. It can be seen that the maximum speed fluctuation of this driver is 15 km/h, the acceleration is −0.45 m/s2, and the speed fluctuation is small, so it can be judged that the driver is smoother when driving the vehicle.

Combined with the above results, it is found that the degree of acceleration and deceleration of different drivers in the driving process is different, so this paper proposes that multi-driver cluster analysis can be carried out. Moreover, the start–stop process of the driver’s way to the intersection is more complicated, so this study does not consider the data that the speed continues to be 0 for a long time.

#### 3.2.2 Trajectory visualization analysis

Every vehicle equipped with a GPS vehicle terminal will have vehicle positioning data in the monitoring platform. The terminal sends positioning data to the platform once at the interval of seconds, and the platform records the data and connects the latitude and longitude points according to the positioning in chronological order to form the running trajectory of the vehicle.

Through the trajectory visualization, the position and driving track of the vehicle on the road can be obtained intuitively. In this paper, we use the folium package in Python to connect the latitude and longitude points into a line, to get the running track of the vehicle. The following figure shows the driving route of a driver for 3 days, it can be seen very intuitively that the 3 days of the driving route were blue route, black route, and red route, of which the black route and red route through the highway and tunnel, and the black line and the red line are partially overlapped. The specific trajectory diagram is shown in Supplementary Figure S1.

#### 3.2.3 Drift data analysis

Vehicles in the driving process will certainly be due to some reasons that lead to the vehicle’s trajectory and the actual road does not match, resulting in incomplete trajectory, or trajectory is a folded line; this phenomenon is known as the vehicle drift phenomenon. This phenomenon is common, and the main reason is the signal propagation process of the error or the impact of weather changes. Generally, paper is also easy to occur when the vehicle is traveling too fast or when it is stationary. Take a high-speed vehicle as an example, the vehicle driving in Hangrui Expressway, and the terminal in the process of uploading data to the platform, there are two consecutive speeds of 0, and the corresponding latitude and longitude data have no change (see Supplementary Table S2), from which it can be judged that the vehicle trajectory has drifted, and it is found that through the visualization of the trajectory to verify that the black line indicates that the vehicle’s trajectory, the vehicle’s trajectory is discounted in this section. The black line indicates the vehicle trajectory, and the vehicle trajectory is discounted in this section and is not on the road, so the vehicle trajectory does drift.

Vehicle drift will produce speed 0 data, which is also one of the reasons why this paper does not choose long-time speed 0 data.

### 3.3 Data preprocessing

The database has more than 15 million data, but these data are not completely available, such as the speed continues to be 0, the existence of drift data, etc., to avoid the selection of this type of data, and it is necessary to pre-process the data. Because of the large amount of data provided, some operations are needed to process the data, which can be sorted, filtered, exported, etc. through Navicat, and Navicat connects to MySQL to perform batch operations on the data. Supplementary Figure S2 below shows the data in Navicat, and SQL language can also be used to query the data and other operations to facilitate data processing.

Due to the confusion of the date of the given data, first of all, the data are imported into Navicat for merging and in chronological order, and then, the SQL query language will upload data with more drivers filtered out; these drivers for the later focus on the object of the study and then finally use the query language according to the date of the respective export excel table for subsequent research.

Given the type of road drivers drive urban roads and highways, if they are put together in the study, will inevitably affect the results of the study, it was decided to distinguish between the types of roads according to the speed interval, according to the general experience of the speed of 60 km/h for the division. After the above operation, this paper screened 12,500 data, of which 6,400 data were for drivers in the 0–60 km/h interval and the remaining 6,100 data for drivers above 60 km/h, and each driver has 100 data.

#### 3.3.1 Box-line chart anomaly detection

GPS data are often interfered with when uploading, resulting in incomplete data or errors; therefore, extra attention needs to be paid to these outliers, and it is very inappropriate to allow the existence of outliers, which will have a bad impact on the data analysis, so outlier detection is needed in the data processing stage.

**Figure 3:**
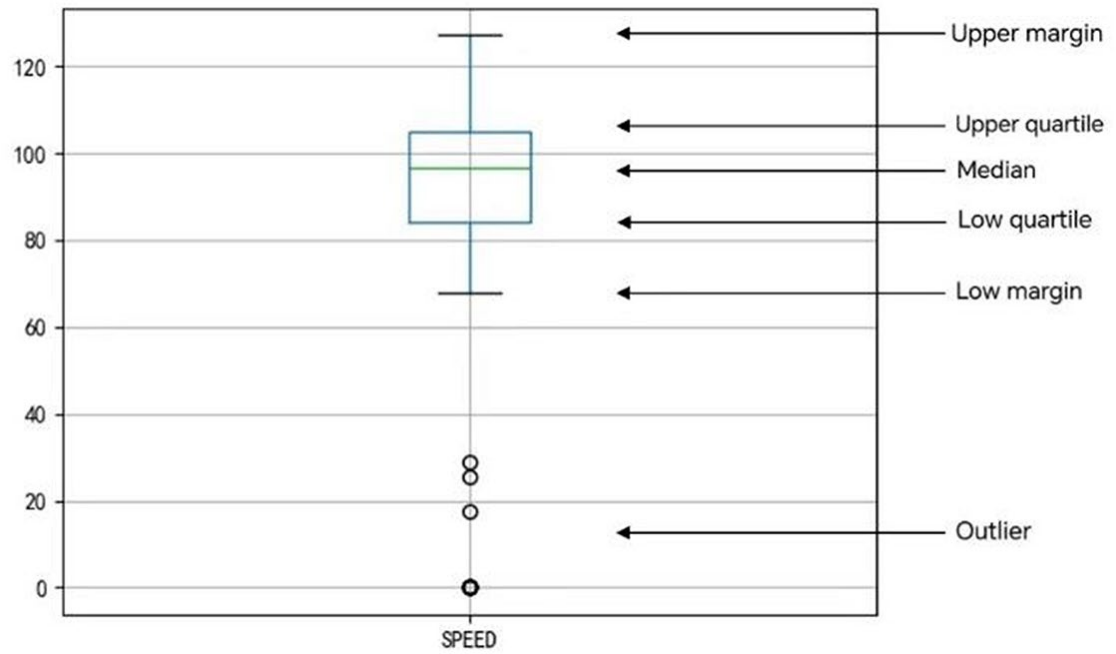
Box plot detection.

In this paper, we use box-and-line diagrams for outlier detection, which can reflect the distribution of a set of data. As shown in Figure 3, taking the speed of a certain time series as an example, the meaning of each line in the graph is indicated in the graph, the top line is the upper edge, the blue line above the rectangular box is the upper quartile, the green line in the rectangular box is the median, the blue line below the rectangular box is the lower quartile, the bottom black line is the lower edge, and the bottom black circle is the outliers.

The following explanations are given for some of the terms:

Lower quartile Q1, median Q2, and upper quartile Q3 are the data arranged from smallest to largest ranked in the 25th, 50th, and 75th percentiles, respectively; quartile distance IQR is the difference between the upper and lower quartiles.

The formulas for the upper and lower margins are as follows (see Equations 1, 2):

(1) upperedge=Q3+1.5IQR

(2) lowermargin=Q1−1.5IQR

The outliers are the data beyond the upper and lower edges.

The box-and-line plot in the chart above reflects the driver’s speed sequence data over a period of time is used as an example for outlier detection. From the results, it can be seen that most of the data are concentrated above 80 km/h, while the detected outliers are below 30 km/h, with more 0 values.

#### 3.3.2 Handling of outliers

For outliers, if they are not handled or directly deleted, it will lead to insufficient data or may also lead to changes in the distribution of data, affecting the subsequent data analysis. Given this, this paper decides to utilize the method of dealing with missing values to deal with outliers. In this paper, with the help of SPSS software, missing values can be detected, according to the missing data and then utilize the linear interpolation method and filled in. Linear interpolation is the process of constructing a linear function using the upper and lower data of the missing values and then utilizing their corresponding function values in the linear function to fill them in. For the time series, the missing value of the speed will have a great correlation with the previous speed and the next speed, but the correlation with the whole speed time series is not strong, so the method is applicable to fill the speed, which is also the reason why this paper did not choose the linear trend interpolation.

Take the speed of a period series as an example, we can see from the analysis results that there are three missing values in 100 data, for the number of extreme values, the same principle as the above box-and-line diagram, the number of lower than the lower edge or more than the upper edge of the number of considered as the number of extreme values, as can be seen from Table 1, at this time, the number of extreme values are all 0, indicating that there are no outliers, and also proves the previous step of the outlier detection of the outlier will be outlier detection, followed by the use of linear trend interpolation, which is the reason why this paper did not choose linear trend interpolation. After that, the linear interpolation function LINT (V) is utilized to linearly interpolate the missing values to fill the missing values.

**Table 1:** Missing value detection and missing value filling.

Missing value detection		*N*	Average	Standard deviation	Deficiencies		Number of extremes	
					Reckoning	Percentage	Low	High
	V	97	104.275	11.313	3	3.0	0	0

Missing value filling		Outcome variables	Missing values replaced	Cases of non-missing values		Number of active cases	Creating Functions
				First	Last		
	1	V	3	1	100	100	LINT(V)

Number of cases outside the range (Q1-1.5IQR, Q3+1.5IQR).

#### 3.3.3 Indicator extraction

Over some time, the driver’s speed is bound to change during the driving process, and a total of 12 indicators are selected in this paper, which is derived from the speed, namely, maximum speed $v_{\text{max}}$ , minimum speed $v_{\text{min}}$ , average speed (total) $\bar{v}$ , the standard deviation of speed (total) $v_{s}$ , minimum acceleration (total) $a_{\text{min}}$ , maximum acceleration (total) $a_{\text{max}}$ , average acceleration (total) $\bar{a}\text{,}$ standard deviation of acceleration (total) $a_{s}$ , mean acceleration (total) $\bar{a}$ , standard deviation of acceleration (total) $a_{s}$ , mean deceleration (negative) $\bar{a}$ -, and standard deviation of deceleration (negative) $a_{s-}$ . The maximum speed and minimum speed can reflect the range of speed change in this time series, the average value can reflect the average driving state during the driver’s driving process, and the standard deviation can reflect the degree of dispersion of speed or acceleration during the driver’s driving process. So the above driving behavior parameters are adopted as the driving behavior indicators in this paper.

(1) Average speed (total) $\bar{v}$ (see Equation 3), speed standard deviation (total) $v_{s\text{.}}$ (see Equation 4).

(3) $$\bar{v}=\frac{1}{n}\overset{n}{\underset{i=1}{\sum }}v_{i}$$

(4) $$v_{s}=\sqrt{\frac{1}{n}\overset{n}{\underset{i=1}{\sum }}{\left(v_{i}-\bar{v}\right)}^{2}}$$

where $v_{i}$ is the ith speed uploaded by the vehicle; n is the sample size of the data; $\bar{v\;}$ is the average speed; and $v_{s}$ is the standard deviation of the speed.

(2) ean acceleration (total) $\bar{a}$ , standard deviation of acceleration (total) $a_{s}$


(5) $$a_{i}=\frac{v_{m}-v_{m-1}}{t_{m}-t_{m-1}}$$

(6) $$\bar{a}=\frac{1}{n}\overset{n}{\underset{i=1}{\sum }}a_{i}$$

(7) $$a_{s}=\sqrt{\frac{1}{n}\overset{n}{\underset{i=1}{\sum }}{\left(a_{i}-\bar{a}\right)}^{2}}$$

where $t_{m-1}\;\mathrm{and}\;v_{m-1}$ are the time and speed at the previous moment; $t_{m}\;\mathrm{and}\;v_{m}$ are the time and speed at the next moment; $a_{i}$ is the speed of the vehicle; n is the sample size of the data; $\bar{a\;}$ is the mean acceleration; and $a_{s}$ is the standard deviation of the acceleration (see Equations 5–7).

This metric is for the total sample of data and is distinguished from the following metrics.

(3) Mean acceleration (positive) ${\bar{a}}_{+}$ (see Equation 8), standard deviation of acceleration (positive) $a_{s+}$ (see Equation 9)

(8) $${\bar{a}}_{+}=\frac{1}{n}\overset{n}{\underset{i=1}{\sum }}a_{i+}$$

(9) $$a_{s+}=\sqrt{\frac{1}{n}\overset{n}{\underset{i=1}{\sum }}{\left(a_{i+}-{\bar{a}}_{+}\right)}^{2}}$$

where $a_{i+}$ is the positive acceleration in the sample; ${\bar{a}}_{+}$ is the mean value of positive acceleration in the sample; and $a_{s+}$ is the standard deviation of positive acceleration in the sample.

(4) Mean deceleration (negative) ${\bar{a}}_{-}$ (see Equation 10), standard deviation of deceleration (negative) $a_{s-}$ (see Equation 11).

(10) $${\bar{a}}_{-}=\frac{1}{n}\overset{n}{\underset{i=1}{\sum }}a_{i-}$$

(11) $$a_{s-}=\sqrt{\frac{1}{n}\overset{n}{\underset{i=1}{\sum }}{\left(a_{i-}-{\bar{a}}_{-}\right)}^{2}}$$

Same as (3) explanation.

For the maximum velocity $v_{\text{max}}$ , the minimum velocity $v_{\text{min}}$ , the minimum acceleration (total) $a_{\text{min}}$ , and the maximum acceleration (total) $a_{\text{max}}$ will not be repeated.

## 4 Driving behavior analysis based on trajectory data

### 4.1 Indicator data processing

According to the principle of indicator extraction in the previous chapter, this paper obtains a total of 12,500 data strips and selects a total of 64 drivers with speeds in the interval of 0–60 km/h and a total of 61 drivers with speeds in the interval of more than 60 km/h, with 100 data strips for each driver, and it is important to note that the selected data are all consecutive time series and the speeds do not continue to 0 for a long period. The final collation of the results is as follows in Table 2. The final results are summarized in Supplementary Table S3.

**Table 2:** Evaluation results for drivers at speeds above 60 km/h.

Methodologies	Evaluation indicators		Running time
	Contour Coefficient	CH	
K-MEANS	0.78	359	1.75 s
K-MEDOIDS	0.60	257	1.93 s
K-shape	0.76	324	2.43 s

### 4.2 Principal component analysis

Since all 12 indicators are centered around speed and acceleration, there is bound to be some correlation among them. To ensure accurate clustering, it is necessary to downscale them and convert these indicators into uncorrelated variables. In this paper, the principal component analysis method is used, through the analysis of the KMO measure value of 0.72, the measure value greater than 0.7 can be considered that the selected 12 indicators have correlation, so it is necessary to carry out the dimensionality reduction process so that it is converted into a few major components as the new indicators.

**Figure 4:**
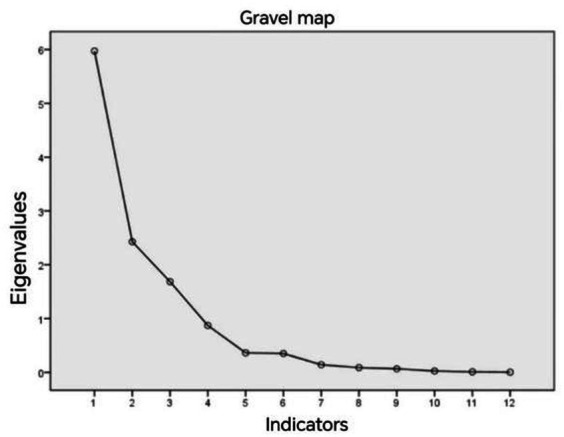
Gravel diagram.

According to the eigenvalues in the fragmentation diagram, the eigenvalues of the first three indicators are all greater than 1, so the first three principal components are selected to replace the original 12 indicators, and the results of the fragmentation diagram are shown in Figure 4.

Supplementary Table S4 shows the principal component analysis table for drivers with speeds of 60 km/h or more. From the figure, it can be seen that both P1 and P2 in the matrix before rotation are related to acceleration and P3 is related to velocity, but it does not differentiate between acceleration and deceleration, so the matrix is rotated. From the rotated matrix, it can be seen that R1 is related to deceleration, R2 is related to acceleration, and R3 is related to velocity.

### 4.3 Driving behavior pattern classification based on clustering

Before clustering, the Hopkins statistic is used to determine whether the data used can be analyzed by clustering; if the value of this statistic is 0.5, it is considered that the data used are uniformly distributed, and the clustering trend is not strong, and it is not recommended to carry out the clustering; if the statistic is at [0.7, 0.99], it means that the data clustering trend is better, and the clustering analysis can be carried out (Banerjee, 2004; Wright, 2022). It is verified that the speed of this paper is 0–60 km/h and 60 km/h above the two types of data, Hopkins statistic value by the Supplementary Table S5 can be obtained above 0.8, so it can be clustered.

This paper takes two classic clustering algorithms K-MEANS and K-MEDOIDS, these two algorithms are widely used, have simple principles, easy to implement, for the two algorithms’ clustering results, this paper according to the clustering indexes selected clustering effect is good algorithms as this paper’s driving pattern clustering analysis method. The following briefly introduces the principle and realization process of the two algorithms.

#### 4.3.1 Driving pattern segmentation based on K-MEANS clustering

The K-MEANS clustering algorithm is an unsupervised learning algorithm. K-MEANS clustering algorithm is an unsupervised learning algorithm. The following are the general steps of the K-means algorithm:

Step 1: Initialization: randomly select K centers of mass (centroids), and K samples can be randomly selected from the dataset as initial centers of mass.

Step 2: Assign samples: for each sample point, calculate the distance to each center of mass and assign it to the cluster represented by the closest center of mass.

Step 3: Update the center of mass: for each cluster, calculate the mean (i.e., the center of mass) of all sample points and update the center of mass to the new mean.

Step 4: Repeat steps 2 and 3 until the center of mass no longer changes or the maximum number of iterations is reached.

Step 5: Output: return the cluster to which each sample belongs.

For easy understanding, its steps are described in this culture, m drivers are randomly selected as the clustering center in the data, m is the number of categories of driving patterns, and then, the distance between each driver curve and these m driver curves is calculated separately; the clustering center with the shortest distance is selected and grouped with it. Every time the clustering center gets driver data, all the curves in the class are recalculated based on the average value, so the clustering center is not the actual driver curve of the data but the calculated curve. This is an iterative process until the clustering results no longer change that iteration is complete.

**Figure 5:**
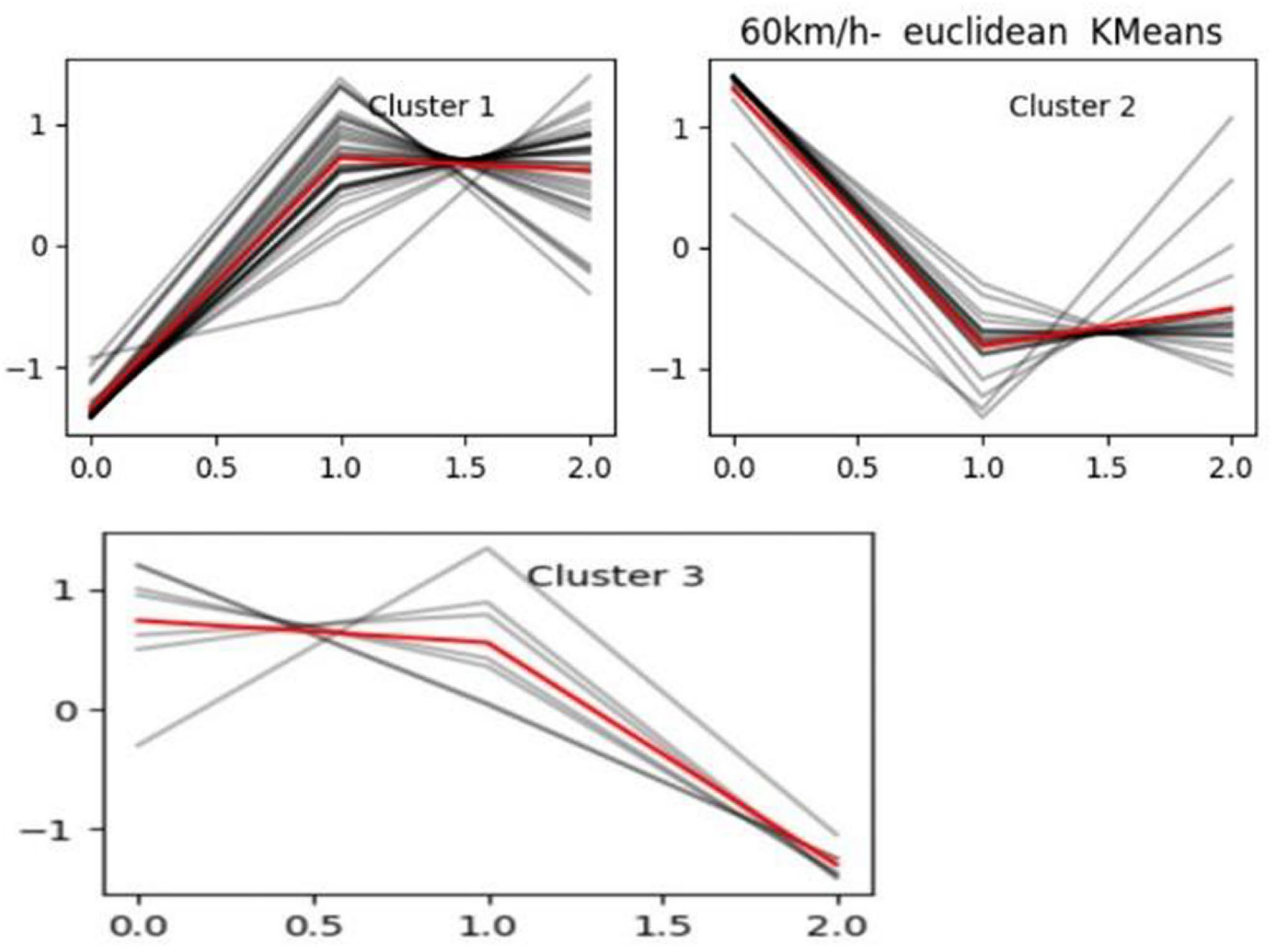
Speed 0–60 km/h driver *K*-means clustering.

There are various distance metrics chosen for clustering algorithms, and the Euclidean distance is used in this paper. According to the principal components of the principal component analysis after the dimensionality reduction of the curve clustering, similar driving patterns of drivers clustered into a class, the following Figure 5 for the speed of 0–60 km/h driver driving pattern clustering effect.

The final clustering results obtained are shown below Table 3.

**Table 3:** K-MEANS clustering.

Speed 0-60km/h driver		Drivers with speeds above 60km/h	
Form	Serial number	Form	Serial number
I	2, 8, 9, 13, 15, 17–19, 21–26, 28, 30, 31, 33, 37, 39, 41, 43, 45, 46, 48, 52-58, 60, 61, 63, 64	I	2, 3, 5, 6, 8–11, 14–16, 19–22, 25, 34–36, 42, 44, 46–48, 54, 56, 58–61
II	1, 4–7, 10, 11, 14, 27, 29, 32, 34, 35, 38, 40, 42, 44, 47, 49–51	II	1, 4, 7, 12, 13, 17, 18, 23, 24, 26–28, 30–32, 37–41, 43, 49–53, 55
III	3, 12, 16, 20, 36, 59, 62	III	29, 33, 45, 57

#### 4.3.2 Driving pattern segmentation based on K-MEDOIDS clustering

The K-MEDOIDS algorithm is very similar to the K-MEANS algorithm, with the difference that the K-MEANS algorithm uses the mean of the curves in the class as a reference, which may not be the actual line that exists, whereas, the K-MEDOIDS algorithm uses the middle-most line in the size of the curves in the class as the clustering center.

The basic idea of K-Medoids algorithm is to divide the dataset into K different clusters, each with a representative sample called Medoid. Unlike K-Means algorithm, K-Medoids algorithm restricts the Medoid to the actual sample points in the dataset rather than an arbitrary virtual center of mass.

K-Medoids is an algorithm used for cluster analysis, and it is one of the division-based methods. It is similar to the K-Means algorithm but employs a different strategy in selecting the center of mass.

The steps of the K-Medoids algorithm are as follows (Huang et al., 2016; Nurhayati et al., 2018):

Step1: Randomly select K sample points as the initial Medoid.

Step 2: Assign each sample to the cluster represented by its nearest Medoid.

Step 3: For each cluster, calculate the sum of the distances from all samples to all other samples within that cluster and select a new Medoid to minimize the sum of the distances.

Steps 2 and 3 are repeated until the cluster no longer changes or the maximum number of iterations is reached.

The advantage of the K-Medoids algorithm is that it is robust to noise and outliers because the Medoid consists of actual sample points that better represent the characteristics of the cluster. However, the computational complexity of the K-Medoids algorithm is high because the sum of distances needs to be computed in each iteration. The clustering results are shown in Table 4.

**Table 4:** K-MEDOIDS clustering.

Speed 0–60 km/h driver		Drivers with speeds above 60 km/h	
Form	Serial number	Form	Serial number
I	2, 8, 15, 21, 23, 25, 33, 37, 43, 45, 55, 57, 58, 61, 62, 64	I	2, 3, 6, 10, 11, 14, 16, 19, 20, 22, 25, 34, 36, 44, 46, 47, 54, 56, 68
II	9, 13, 17-19, 22, 24, 26, 28, 30, 31, 39, 41, 46, 48, 52–54, 56, 60, 63	II	1, 4, 7, 12, 13, 17, 18, 23, 24, 26–28, 30–33, 37–41, 43, 49–53, 55, 57
III	1, 3–7, 10–12, 14, 16, 20, 27, 29, 32, 34–36, 38, 40, 42, 44, 47, 49–51, 59	III	5, 8, 9, 15, 20, 29, 35, 42, 45, 48, 59–61

### 4.4 Analysis of clustering results

The drivers have been clustered using two clustering algorithms, to better analyze the clustering results, this paper measures the advantages and disadvantages of the clustering algorithms by two evaluation indexes, namely, the contour coefficient and CH, and the two indexes are described below.

#### 4.4.1 Contour coefficient

In this paper, the contour coefficient describes the similarity of a particular driver to the similarity between the driver’s class and other classes. The range of the coefficient is [−1,1], and the closer the value to 1 indicates that the relationship between the driver and his class fits better, i.e., if the value of the coefficient is higher, the better the clustering is, and if the value is lower, it indicates that the clustering effect is not good, which may be that the number of categories is not selected ideally or the data are not suitable for clustering by this method.

(12) $$SIL=\frac{b-a}{\max\left(a,b\right)}$$

where SIL (see Equation 12) is the contour coefficient; a is the average distance between the driver and other drivers in the class; and b is the average distance between the driver and drivers in other classes.

#### 4.4.2 CH

The smaller the sum of squares of the distances within the class, the better, and the larger the sum of squares of the distances between the classes, the better, as a measure of its tightness and separation. The larger the value of CH (see Equation 13) represents the better the clustering effect.

(13) CHk=trBKk−1trWkn−k

where n is the number of clusters; k is the current class; $tr\left(B_{K}\right)$ is the trace of covariance matrix between classes; and $tr\left(W_{k}\right)$ is the trace of covariance matrix within classes.

Combining the calculation process of the above two indicators, the evaluation results of drivers with speeds 0–60 km/h and above 60 km/h (see Table 5) can be obtained. In addition, when evaluating the indicators, a clustering algorithm k-shape based on shape similarity is added, which is validated as a research method together with K-MEANS and K-MEDOIDS. From the table, it is obvious to see that the values of the two evaluation indexes contour coefficient and CH of K-MEANS are higher than those of K-MEDOIDS, which indicated that the clustering effect of the K-MEANS algorithm is better, and it is more suitable for the analysis of driving patterns in this paper. While the k-shape algorithm although its contour coefficient is similar to K-MEANS, the CH indicator still has some differences; this is due to the fact that k-shape is a shape-based time-series clustering algorithm for the one-dimensional dataset, while this paper is for the multi-dimensional dataset. In addition to the three methods in terms of running time, K-MEANS is shorter.

**Table 5:** Driver evaluation results.

Speed 0–60 km/h driver				Drivers at speeds above 60 km/h			
Methodologies	Evaluation indicators		Running time	Methodologies	Evaluation indicators		Running time
	Contour coefficient	CH			Contour coefficient	CH	
K-MEANS	0.70	222	1.71s	K-MEANS	0.78	359	1.75s
K-MEDOIDS	0.57	176	1.95s	K-MEDOIDS	0.60	257	1.93s
k-shape	0.70	164	2.45s	k-shape	0.76	324	2.43s

Fixing a certain method, these two types of drivers with speeds of 0–60 km/h and above 60 km/h are observed. From their evaluation indexes, it can be seen that those above 60 km/h have higher evaluation indexes than 0–60 km/h, i.e., the clustering effect is better, this is because drivers driving on highways have their speeds more than 60 km/h, with less speed fluctuation, while drivers driving 0–60 km/h drive on urban roads, where there are more and more complex factors affecting driver behavior, such as pedestrian, non-motorized vehicles, and speed limit. Therefore, the speed fluctuation of vehicles on urban roads is larger, and each index is also affected by it, so the clustering effect is not as good as that above 60 km/h.

### 4.5 Driver driving state analysis

This paper clusters drivers from a macro-point of view according to driving behavior indicators and obtains drivers of three driving modes. However, in practice, drivers will have different driving behaviors when encountering different traffic conditions, i.e., drivers will exist in uniform speed, acceleration, deceleration, and other states. Based on the above clustering results, one of the drivers is selected in this section for micro-analysis of their driving state, and the method chosen here is a combination of PAA and SAX. As time-series data, the extracted features need to be reasonably simplified to reduce the complexity. In the process of method exploration, this paper uses the symbol aggregation approximation method to convert the data into simplified symbols. The symbol aggregation approximation algorithm was proposed by Lin et al. (2003), which can convert the input time series into strings, such as ‘back.’

The SAX process consists of three steps.

(1) Normalize the time-series data;

(2) Converting the time series of length n to the time series of length w by using PAA dimensionality reduction, the PAA dimensionality reduction formula is as follows (see Equations 14, 15):

(14) $$j=\frac{n}{w}\left(i-1\right)+1$$

(15) $$\bar{c_{i}}=\frac{w}{n}\overset{\frac{n}{w}i}{\underset{j}{\sum }}c_{j}$$

where $c_{j}$ is the original time series of length n , and $\bar{c_{i}}$ is the sequence of length w after dimensionality reduction;

(3) Convert to string according to PAA data.

**Figure 6:**
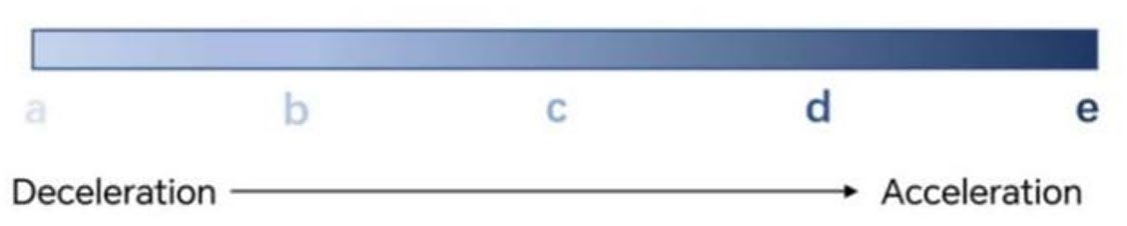
Schematic diagram of the five state changes.

In this paper, the acceleration of 50 consecutive time points is selected, and 25 data points can be obtained by using PAA to take the mean value of every two data points and then using SAX symbolic representation; the first must be delineated as an odd number of states to find the middle level for the neutral state (uniform speed state), and the acceleration and deceleration states on both sides, respectively; if it is delineated as three states, each state has a large range of thresholds, which is not detailed enough. Therefore, this paper finally divides the individual driver’s driving behavior into five classes, i.e., five states, from deceleration state to acceleration state according to the color scale change visualization as shown in Figure 6:

- stage of greater deceleration
- stage of smaller deceleration
- close to uniform velocity stage
- smaller acceleration stage
- stage of larger acceleration.

**Figure 7:**
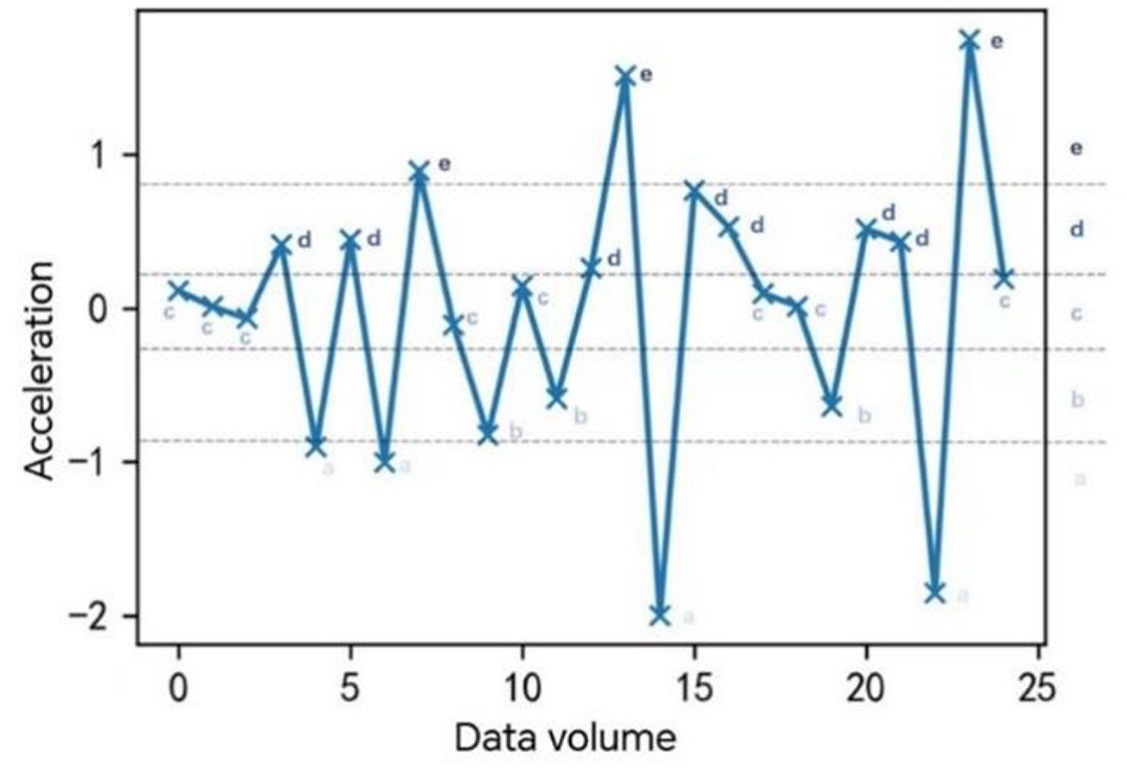
Individual driver driving state effect.

Taking a certain driver with a speed of 60 km/h or more as an example, the driving behavior state (Figure 7) is as follows:

## 5 Based on driving behavior-based traffic emission analysis

### 5.1 VSP-based vehicle motion characterization

Vehicles driving in the road, subject to the influence of external factors, such as pedestrians, other vehicles, signal control, and weather conditions, will make the vehicle’s operating state change, so the vehicle power changes and power and emissions are closely related to each other, which in turn will produce different emissions. Jiménez-Palacios first put forward the concept of specific power (Jiménez-Palacios, 1999), which is used to characterize the vehicle’s operating conditions and power linkage. He believed that specific power can link the relationship between vehicle state and emissions. The specific power of a motor vehicle is the power per unit time per unit mass of the motor vehicle in kW/t or m^2^/s^3^, and it represents the sum of the power of all the forces that impede the forward movement of the vehicle (see Equation 16).

(16) $$VSP=\frac{\frac{d}{dt}\left(KE+PE\right)+F_{\mathrm{rolling}\;.\;}v+0.5\rho_{a}\;.\;C_{D}\;.\;A\;.\;{\left(v+v_{w}\right)}^{2}\;.\;v}{m}$$

where $\frac{d}{dt}\left(KE+PE\right)$ is the power consumed by kinetic and potential energy; $F_{\mathrm{rolling}}$ is the rolling resistance; $0.5\rho_{a}\;.\;C_{D}\;.\;A\;.\;{\left(v+v_{w}\right)}^{2}$ is the air resistance; and m is the mass of the vehicle.

According to the analysis of the vehicle’s kinematic performance, the vehicle’s emission is closely linked to the engine output, i.e., power. The power variation of the vehicle has an important relationship with the vehicle speed. This specific power model can correlate the vehicle emissions with the vehicle operating conditions and accurately quantify the vehicle emissions. The speed in the GPS data is the raw value, so the error of using the speed for the study is less. Moreover, the above formula involves more variables, which are sometimes not directly accessible, and for general light-duty vehicles, a simplified formula can be used (see Equation 17):

(17) $$VSP=v�\left(1.1�a+9.8\mathrm{at}+0.132\right)+0.000302�v^{3}$$

where v is in m/s, and a is in m/s^2^.

### 5.2 Calculation of traffic emissions based on MOVES

Traffic emissions are affected by several parameters, such as road type, major emission sources on the road, fuel type, etc., but in view of the inconvenience of obtaining other parameters, this paper is mainly calculated with the help of speed parameters. Although parameters such as speed, temperature, fuel type, and road type have an impact on the MOVES model, speed is the most dominant parameter, and other parameters such as temperature and fuel type are used as parameters for adjustment.

The MOVES emission rate establishes a direct relationship with the VSP, and a large number of studies have proved that the vehicle VSP can characterize the emission rate better than the speed and acceleration, so this paper is simplified to use the speed and acceleration to calculate the specific power VSP of the vehicle, so that the calculated VSP value can be used by the Supplementary Table S6 will be the emission rate and the VSP establishes a direct relationship.

### 5.3 Model validation

This section analyzes drivers with speeds of 0–60 km/h and speeds of 60 km/h or more, respectively. GPS data are uploaded at second intervals, some data are uploaded at about 30s, and some data are uploaded at intervals that are too large or too small. Due to the differences in the uploading intervals of GPS data, this results in the total time occupied by the time-series data being different for each driver. To avoid errors when analyzing drivers’ driving patterns and traffic emissions, this paper uniformly converts all time-series data into emissions produced per unit of time (per unit of hour) when calculating emissions using the time-series data.

Based on the above clustering results, this paper, after obtaining the emissions per unit of time of each driver, calculates the mean value of the emissions of all drivers in each category of driving mode, which represents the pollutant emissions of the drivers in that category. In this paper, we did not adopt randomly selecting a driver in each driving mode to represent the degree of emissions of the driving mode because the method is more random and cannot well characterize the degree of emissions of the driving mode. The results of the final traffic emission calculations are shown in Table 2. As a whole, among the four emissions, CO2 emissions are much higher than other emissions, and CO2 emissions account for more than 96% of the total emissions, although CO2 is not a pollutant, as a greenhouse gas, its excessive emissions will inevitably have a serious harm to the environment. NOX and HC are relatively small compared with CO2, and they account for approximately 1.5% of the total emissions. In the analysis of driving modes and emissions, for drivers with speeds of 0–60 km/h, the emissions of each driving mode are reduced from Class I to Class III driving modes according to the clustering results; for drivers with speeds of more than 60 km/h, the emissions of Class I driving modes are the highest, the emissions of Class III driving modes are the lowest, and the emissions of Class II driving modes are located in the range of Class I and Class III driving modes. The emissions of drivers in driving mode II are located between those of drivers in driving mode I and those in driving mode III. In addition, comparing the two tables, it is found that the degree of emissions from the driving mode of drivers with speeds above 60 km/h is higher than that of drivers with speeds between 0 and 60 km/h. This is because although the acceleration of drivers traveling on highways is not as varied as that of drivers on urban roads, the speeds are much higher than those of drivers on urban roads, which leads to differences in calculating the VSP, which results in different emissions.

The method adopted in this paper in calculating emissions is to calculate the average emissions of drivers in this category of driving modes for each driving mode, to avoid too much neutralization of data, this paper analyzes the degree of dispersion of the emission data of this category of driving modes to determine whether it is appropriate to use the average to characterize the driving modes, only the degree of dispersion of pollutants is statistically determined here, and CO2 is not taken into account. Taking drivers with speeds of 0–60 km/h as an example, the standard deviation is used as a measure of the dispersion degree of drivers, and the following statistical characteristics are obtained as shown in Table 6.

**Table 6:** Emission table for driving modes.

Drivers with speeds of 0-60km/h	Driving mode	Emission(g/h)				Total emissions
		CO	CO2	NOX	HC	
	I	331.27	12670.00	16.73	7.51	13025.50
	II	253.12	11456.06	14.06	6.59	11729.82
	III	167.47	9957.92	10.92	5.50	10141.81
**Drivers with speeds of 60km/h or more**	**Driving mode**	**Emission(g/h)**				**Total emissions**
		**CO**	**CO2**	**NOX**	**HC**	
	I	403.11	13299.68	18.45	8.20	13729.44
	II	335.10	12887.62	17.15	7.61	13247.49
	III	392.45	13204.50	18.17	8.09	13623.22

From the table, it can be seen that the standard deviation of NOX and HC is very small, and through the rule of mean plus minus standard deviation, it can be found that its range corresponds roughly to its corresponding degree of emission, while the standard deviation of CO has a larger value compared with the other two pollutants, and there is a partial overlap in the range of the values in Table 7 using the rule of mean plus minus standard deviation, due to the difference in speed and actual speed caused by the difference in the intervals of the return data of the GPS data itself. This is due to the GPS data itself return data interval caused by different speeds and the actual difference, and the use of the average value cannot be a fine delineation of the range but can be compared to the emissions of the approximate degree of emissions.

**Table 7:** Analysis of degree of dispersion.

Driving mode	(Statistics) standard deviation		
	CO	NOX	HC
I	152.50	3.96	1.52
II	105.15	3.76	1.28
III	24.77	1.27	0.35

The above is a macroanalysis of the relationship between driving patterns and traffic emissions. Next, we will analyze the relationship between each driving state and traffic emissions from a micro-perspective. The results calculated in the table below are the emissions produced per unit time (s), i.e., the emission rate. For drivers, it is obvious from Table 8 that the pollutant emissions are the largest if the vehicle is in a state of high acceleration. The pollutant emissions are the highest if the vehicle is in the state of high acceleration. According to the color scale change, the pollutant emission decreases sequentially until the state of smaller deceleration b. For the state of larger deceleration a, the total emission rate is still decreasing, although there is a slight increase in CO, NOX, and HC.

**Table 8:** Individual driver emission rates by status.

Driving mode	Emission rate(g/s)			
	CO	CO2	NOX	HC
a	0.0506	2.4871	0.0029	0.0016
b	0.0152	2.9165	0.0026	0.0013
c	0.0482	3.8003	0.0045	0.0019
d	0.0907	5.0618	0.0079	0.0028
e	0.2930	5.9483	0.0108	0.0043

In summary, this paper has clustered the driving patterns of drivers and obtained the relationship between various driving patterns and traffic emissions. Next, the decision tree method is used to predict drivers with unknown driving patterns to obtain their corresponding pollutant emission levels.

Based on the above clustering results, the driving pattern of each driver is obtained and the driver is manually labeled for the decision tree prediction. For example, a driver was randomly selected from the database and the data were processed as described in the previous sections and predicted using the decision tree based on 12 indicators. The metrics for this driver are shown in Supplementary Table S7.

The decision tree is visualized in *VS* Code with the following results in Supplementary Figure S3.

After the decision tree prediction, it can be obtained that the driver’s driving mode is Class I. Since this driving mode emits more emissions, it can be suggested that the driver should not accelerate and decelerate frequently and should standardize his driving.

## 6 Conclusion

Based on the GPS track data, this paper clusters the driving patterns by unsupervised and supervised machine learning algorithms and also divides the driving status of individual drivers, and the research work and conclusion of this paper are specifically summarized as follows:

1. In the data processing stage, this paper first divides drivers into two categories with a speed of 60 km/h and subjectively believes that drivers with 0–60 km/h drive on city roads, and those with more than 60 km/h drive on highways, which avoids the inaccuracy of clustering of drivers in a wide range.
2. This paper analyzes driving behavior from two perspectives. First, from the macro-perspective of the driver’s driving pattern clustering, first of, the use of principal component analysis to reduce the dimension of the 12 indicators, and then two classic clustering algorithms K-MEANS and K-MEDOIDS, according to the Euclidean distance will be divided into three classes, to profile coefficients and other indicators as a judgment condition for the clustering algorithms to be selected, and ultimately selected the K-MEANS; the second is from the micro-perspective of the driver’s driving status analysis. The driver’s driving state was analyzed, and a combination of PAA and SAX was used to represent the driving state as a symbolic image.
3. In this paper, the latest MOVES was chosen as the traffic emission model, and the relationship between traffic emission and driving behavior was analyzed from these two perspectives. Based on the clustering results of driver driving patterns and the division results of driver driving states, the used driver speed, acceleration, and time interval are substituted into the emission model, and the calculated results are converted into the emissions per unit time, from which the relationship between driving behavior and traffic emissions can be obtained. In addition, the decision tree method can be used in this paper to predict the driver of the positional driving mode to estimate the degree of pollutant emission of this driver.

In this paper, based on the trajectory data, the clustering analysis and prediction of multi-driver driving patterns, as well as the symbolic representation of each driving state of individual drivers, and their relationship with traffic emissions are obtained, and the research results have been achieved the expected purpose, but there are still many shortcomings:

(1) Limitations of the number of driver samples

In this paper, in screening GPS data, only the continuous time series whose speed is not 0 is considered, so the number of useful time series obtained in the end is relatively small, which may lead to inaccuracies in cluster analysis or prediction of driving pattern categories due to the insufficient amount of data. Afterward, the study can be considered more comprehensive, not only for the data with non-zero speeds but also for the traffic emissions within the intersection, as appropriate.

(2) Limitations of driving behavior parameters

Only driving behavior parameters, i.e., speed and acceleration, were considered in the principal component analysis and cluster analysis, and the selected indicators were derived from speed and acceleration. Considering more driving behavior parameters, such as headway or headway spacing, may make the clustering results more accurate.

(3) Limitations of GPS data upload interval

Although GPS data are uploaded at second intervals, the intervals are more random and not uniform enough, so they need to be converted into emissions per unit of time when calculating emissions, which may differ from the actual emissions.

(4) Limitations of influencing factors of traffic emission

Traffic emissions are not only related to speed and acceleration but also related to road type, vehicle category, fuel type, etc. In this paper, only the first two factors are considered, and the rest of the factors are not considered. As a result, there may be errors in the calculation of pollutant emissions. The influencing factors can be obtained through other ways in future studies to make the emission calculation more accurate.

## Data availability statement

The original contributions presented in the study are included in the article/Supplementary material, further inquiries can be directed to the corresponding author.

## Author contributions

XD: Conceptualization, Data curation, Formal analysis, Investigation, Software, Writing – original draft. XK: Conceptualization, Investigation, Methodology, Supervision, Writing – review & editing. YG: Conceptualization, Formal analysis, Project administration, Validation, Writing – review & editing. XW: Data curation, Formal analysis, Writing – review & editing.
