# Supplementary material for: Driving behavior characterization and traffic emission analysis considering the vehicle trajectory
Source: Front Psychol. 2024 Jan 29;14:1341611. doi: 10.3389/fpsyg.2023.1341611 (PMC10860677; doi:10.3389/fpsyg.2023.1341611)
Supplement: Supplementary file 1 [file Data_Sheet_1.pdf]

Supplementary Material

1. Supplementary Figures

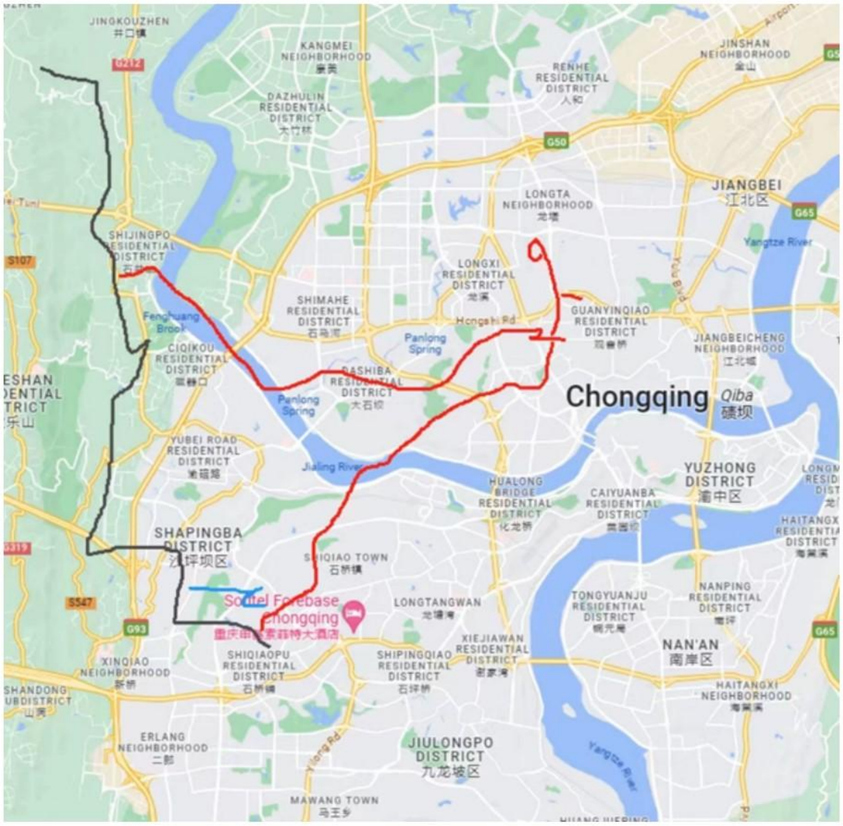

Figure. S1 Trajectory diagram

CAR_ID	LONGITUDE	LATITUDE	SPEED	KMI	HEADING	TIME	RECEIVE_TIME
05b0d5b9c9764e67a3c0c0b4fe1b10bf	106.528768	29.59173	13.4	13	160.30000	2015-09-02 00:01:37	2015-09-02 00:01:33
00a556f047d511e5000098e19087acff	103.98822	30.636588	0	0	0	2015-09-02 00:01:37	2015-09-02 00:01:33
00052569d9a644f68bb022cd0c1c8ac5	105.903065	29.278668	52.9000	52	14.2	2015-09-02 00:01:38	2015-09-02 00:01:34
06088704911449c58e15a384370c8bfb	102.058075	30.556798	13.9	14	354.70001	2015-09-02 00:01:00	2015-09-02 00:01:35
06088704911449c58e15a384370c8bfb	102.057973	30.556821	14.8	16	11.2	2015-09-02 00:01:02	2015-09-02 00:01:36
06624c56cef349f69948adca9c0bb56b	107.888066	27.224536	0	0	96.199997	2015-09-02 00:01:21	2015-09-02 00:01:35
00a76f9837d34000b2c6a3875f77cad8	106.538775	29.575258	0	0	146.80000	2015-09-02 00:01:15	2015-09-02 00:01:35
06824651436603089738	106.862718	28.628013	32.0999	0	339.60000	2015-09-01 23:48:09	2015-09-02 00:02:25
02f7d2511b6d41298887b5edfecca4f5	106.351203	29.376241	0	0	0	2015-09-02 00:02:12	2015-09-02 00:02:26
0380a6cacf0a422292b40d6448270f47	106.62136	29.496165	52.5	51	315.5	2015-09-02 00:02:30	2015-09-02 00:02:26
0805ca3db5ae44f781c68062259532af	106.51931	29.624218	0	0	31.299999	2015-09-02 00:02:26	2015-09-02 00:02:24

Figure. S2 Example of Navicat Data

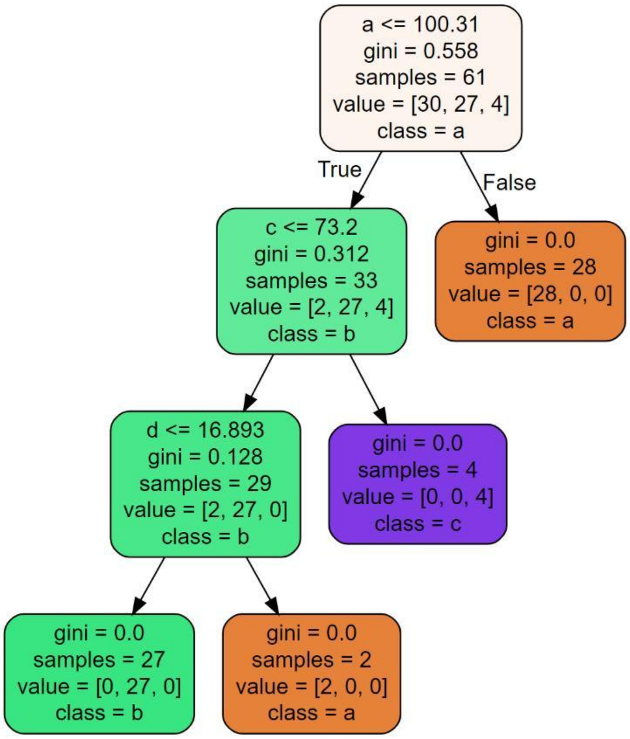

Figure. S3 Decision tree visualization

2. Supplementary Tables

Table S1 Field Descriptions

Field	Explanation of fields	Example
CAR_ID	Vehicles ID	048f66be51d342fcae55cb69bae15d8e
LONGITUDE	Longitudes	119.323258
LATITUDE	Latitude	26.068633
SPEED	Speed	38
KMPH	Obd speed	37
HEADING	Orientations	259.2999878
TIME	Machine time	2023-09-03 00:04:01

RECEIVE_TIME	Platform reception time	2023-09-03 00:04:18

Table S2 Example of data drift

Time	Longitude	Latitude	Speed	Orientations
2015/9/5 11:23:49	105.7564	27.20506	0	240.4
2015/9/5 11:23:54	105.7564	27.20506	0	240.4

Table S3 Driving behavior indicator data

norm / serial number	$\bar{v}$	v_{max}	v_{min}	v_s	$\bar{a}$	a_{max}	a_{min}	a_s	$\bar{a}_+$	$\bar{a}_-$	a_{s-}	a_{s+}
1	19.62	44.50	5.30	9.51	0.05	3.19	-2.19	0.52	0.31	-0.26	0.34	0.49
2	28.47	56.20	7.30	13.13	-0.03	1.56	-2.36	0.51	0.28	-0.35	0.49	0.31
3	21.37	52.50	5.70	10.27	0.06	2.56	-1.18	0.44	0.32	-0.24	0.23	0.42
…												
64	30.76	58.10	8.90	11.58	0.07	2.11	-1.10	0.50	0.42	-0.30	0.27	0.41

Table S4 Principal component analysis matrix

Norm	Pre-rotation matrix			Matrix after rotation		
	P1	P2	P3	R1	R2	R3
$\bar{v}$	0.602	0.332	0.624	0.176	0.328	0.850
v_{max}	0.771	0.208	0.417	0.390	0.440	0.682
v_{min}	0.193	0.233	0.868	-0.054	-0.096	0.913
v_s	0.711	-0.103	-0.334	0.591	0.518	-0.104

$\bar{a}$	0.150	0.854	-0.188	-0.485	0.734	0.119
a_{max}	0.701	0.475	-0.247	0.175	0.854	0.134
a_{min}	-0.687	0.623	-0.120	-0.922	0.031	-0.152
a_s	0.943	-0.261	-0.086	0.859	0.454	0.150
$\bar{a}_+$	0.833	0.355	-0.257	0.353	0.862	0.134
$\bar{a}_-$	-0.823	0.372	0.104	-0.851	-0.313	-0.064
a_{s-}	0.762	-0.592	0.079	0.955	0.053	0.148
a_{s+}	0.804	0.427	-0.235	0.282	0.882	0.165

Table S5 Hopkins statistics scale

Pilot	Hopkins
0-60km/h	0.816
60km/h or more	0.829

Table S6 Emission rates under specific power partitioning

VSP Interval	Emission rate（g/s）			
	CO	CO2	NOX	HC
$(-\infty, -2)$	0.0110	1.5437	0.0010	0.0009
[-2,0)	0.0087	1.6044	0.0010	0.0009
[0,1)	0.0047	1.1308	0.0004	0.0008
[1,4)	0.0122	2.3863	0.0016	0.0010

[4,7)	0.0167	3.2102	0.0026	0.0013
[7,10)	0.0233	3.9577	0.0038	0.0017
[10,13)	0.0293	4.7520	0.0051	0.0021
[13,16)	0.0369	5.3742	0.0064	0.0023
[16,19)	0.0495	5.9400	0.0077	0.0028
[19,23)	0.0638	6.4275	0.0099	0.0030
[23,28)	0.1054	7.0660	0.0127	0.0038
[28,33)	0.2478	7.6177	0.0144	0.0046
[33,39)	0.4131	8.3224	0.0156	0.0057
[39, ∞)	0.6247	8.4750	0.0167	0.0072

Table S7 Projected driver data

norm	$\bar{v}$	v_{max}	v_{min}	v_s	$\bar{a}$	a_{max}	a_{min}	a_s	$\bar{a}_+$	$\bar{a}_-$	a_{s-}	a_{s+}
digital	104.71	122.60	66.40	12.30	0.00	0.56	-1.03	0.28	0.17	-0.24	0.26	0.15
